# Jaw reconstruction with vascularized fibular flap: The 11-year experience among 104 patients

**DOI:** 10.1186/s12957-020-01826-7

**Published:** 2020-02-29

**Authors:** Shengjie Shao, Weihong Wang, Biao Xu, Yu Liu, Zhe Zhang

**Affiliations:** Department of Oral and Maxillofacial Surgery, Affiliated Stomatology Hospital of Kunming, Medical University, No. 1088 Mid Hai Yuan Road, Gaoxin District, Kunming, 650106 Yunnan China

**Keywords:** Vascularized fibular osteomyocutaneous flap, Non-vascularized fibular graft, Severe vascular crisis, Jaw defect

## Abstract

**Background:**

A vascularized fibular osteomyocutaneous flap with severe vascular crisis often results in serious consequences. This study aims to examine the clinical effect of non-vascularized fibular graft on patients with severe vascular crisis after reconstruction of the defect jaw with vascularized fibular osteomyocutaneous flap.

**Materials and methods:**

From December 2007 to December 2018, a total of 104 patients with jaw neoplasms that underwent reconstruction with free vascularized fibular flap were retrospectively analyzed; seven of these cases had postoperative vascular crisis during mandibular reconstruction.

**Results:**

Of the seven cases with postoperative vascular crisis, the vascularized fibular flaps in three patients survived completely, thanks to early detection; two cases were completely necrotic and removed in the end, and the remaining two cases had severe vascular crisis after the removal of the soft tissue attached to the fibular flap. The non-vascular fibular grafts were retained regardless of the severe absorption after follow-ups for 25 and 69 months, respectively.

**Conclusions:**

If vascular crisis occurs following jaw reconstruction with a vascularized fibular osteomyocutaneous flap, early re-surgical exploration effectively improves the salvage rate. In addition, when a severe vascular crisis occurs, the vascularized fibular flap can be changed to a non-vascular fibular graft to reconstruct the mandibular defect, thus avoiding the serious consequences resulting from the complete failure of fibular graft.

## Introduction

Vascularized fibular osteomyocutaneous flap has been currently advocated in jaw reconstruction for large defects, which contributes to the restoration of the facial appearance and chewing function of the patient [1–3]. Notably, vascular anastomosis is required in a free tissue flap; therefore, it is of great importance to detect vascular crisis in the anastomotic blood vessels at the early stage, and to carry out surgical exploration and treatment immediately for achieving successful operation. The delayed discovery of vascular crisis or the presence of the more serious vascular crisis may result in complete necrosis of the free transplanted tissue flap, thereby inducing severe consequences [4–9]. In this study, to avoid serious consequences induced by the vascularized fibular osteomyocutaneous flap with severe vascular crisis, the vascularized fibular osteomyocutaneous flaps in two cases with severe vascular crisis were immediately managed using the non-vascularized fibular grafts. As a result, this remedial treatment contributed to achieving certain clinical effects.

## Patients and methods

From December 2007 to December 2018, 104 patients, including 57 males and 47 females with jaw neoplasms, were retrospectively analyzed at the Department of Oral and Maxillofacial Surgery of the Affiliated Stomatological Hospital of Kunming Medical University. This study protocol was approval by the Human Body Science Committee of Kunming Medical University. All patients provided a written form of consent for the use of their medical information. The vascularized fibular bone flap was immediately reconstructed upon the removal of the jaw neoplasm, and it was then fixed using the YZB2.0 craniotomy system titanium plate and screws (Shenzhen Putianyang Medical Instrument Co., Ltd). The age of patients ranged from 15 to 66 years, with an average of 38.6 years. Of all the enrolled 104 patients, 90 had ameloblastomas, 2 had myxomas, 1 had ossifying fibroma, 1 had odontoma, 7 had squamous cell carcinomas, and 3 had osteosarcomas. Among them, 94 cases had neoplasms in the mandible, while the remaining 10 had tumors in the maxilla. The clinical data of these 7 patients are presented in Table 1.

**Table 1:** Clinical features of 7 patients with vascular crisis

Number	Date	Age(years)	Gender	Diagnosis	Location	Perforator flap	Exploration	Reason	Result
1	2010	51	Male	Ameloblastoma	Mandible	No	No	Vascular crisis	Complete necrosis
2	2011	23	Male	Ameloblastoma	Mandible	No	Yes	Venous crisis	Complete necrosis
3	2013	41	Male	Ameloblastoma	Mandible	Yes	Yes	Severe vascular crisis	Nonvascularized fibular
4	2015	29	Male	Ameloblastoma	Mandible	Yes	Yes	Venous crisis	Survived
5	2016	42	Female	Ameloblastoma	Mandible	Yes	Yes	Severe vascular crisis	Nonvascularized fibular
6	2017	28	Male	Ameloblastoma	Mandible	Yes	Yes	Perforator flap crisis	Survived
7	2017	22	Female	Ameloblastoma	Mandible	Yes	Yes	Perforator flap crisis	Survived

**Fig. 1:**
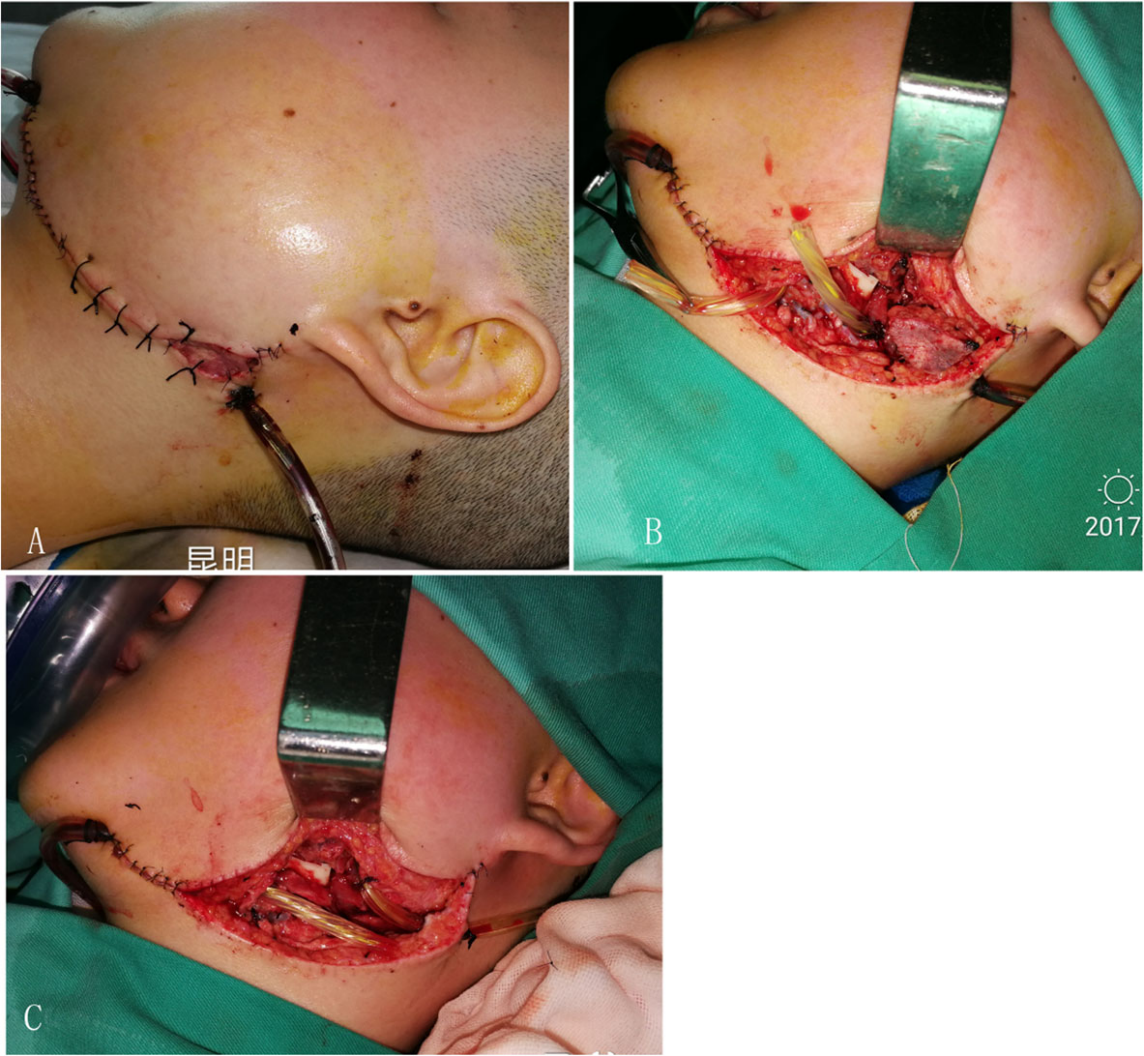
The pedicle of skin paddle was only partially compressed (**a**, **b**), and the perforator flaps were removed (**c**)

**Fig. 2:**
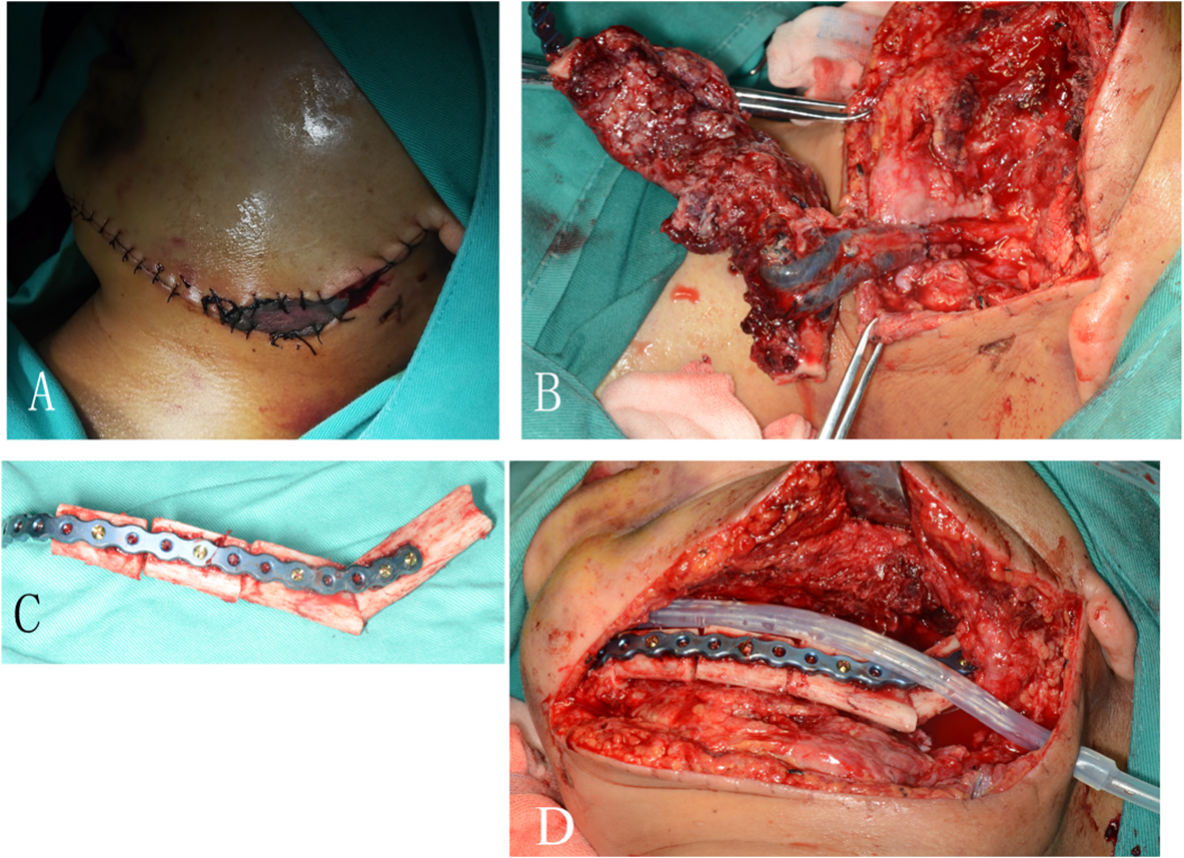
The vascularized fibular osteomyocutaneous flap was extensively congested, which induced a severe arterial and venous crisis (**a**, **b**). All soft tissues attached to the fibular bone including arteries, veins, flexor hallucis longus, and the skin flap were completely removed (**c**). The non-vascularized fibular bone flap and was reattached to the mandibular stump (**d**)

More details of jaw reconstruction are described in our previous reports [10, 11]. Six out of these 7 patients with vascular crisis received immediate surgical exploration under general anesthesia through nasal endotracheal intubation. Afterwards, the wound was washed with a large amount of warm saline, and later the anastomotic artery, vein, fibular bone flap, and fibular perforator vessel were exposed. Two out of these 7 patients with venous crisis received anastomotic incision and re-anastomosis after repeated washing with heparin sodium saline. Moreover, another two patients received partial compression in the perforating vascular pedicle of the skin paddle, and the perforator flaps were removed (Fig. 1). For the remaining two cases, no blood flow was observed after the vein at the anastomotic site was cut. However, the vascularized fibular osteomyocutaneous flap was extensively congested, which induced a severe arterial and venous crisis. Therefore, the fibular bone flaps were removed; meanwhile, all soft tissues attached to the fibular bone including the arteries, veins, flexor hallucis longus, and skin paddle were also completely removed. Subsequently, the vascularized fibular musculocutaneous flap was treated as the non-vascularized fibular graft, which was reattached to the mandibular stump (Fig. 2). Panoramic radiographs or CBCT examinations were performed on these cases during the follow-up period.

## Results

**Fig. 3:**
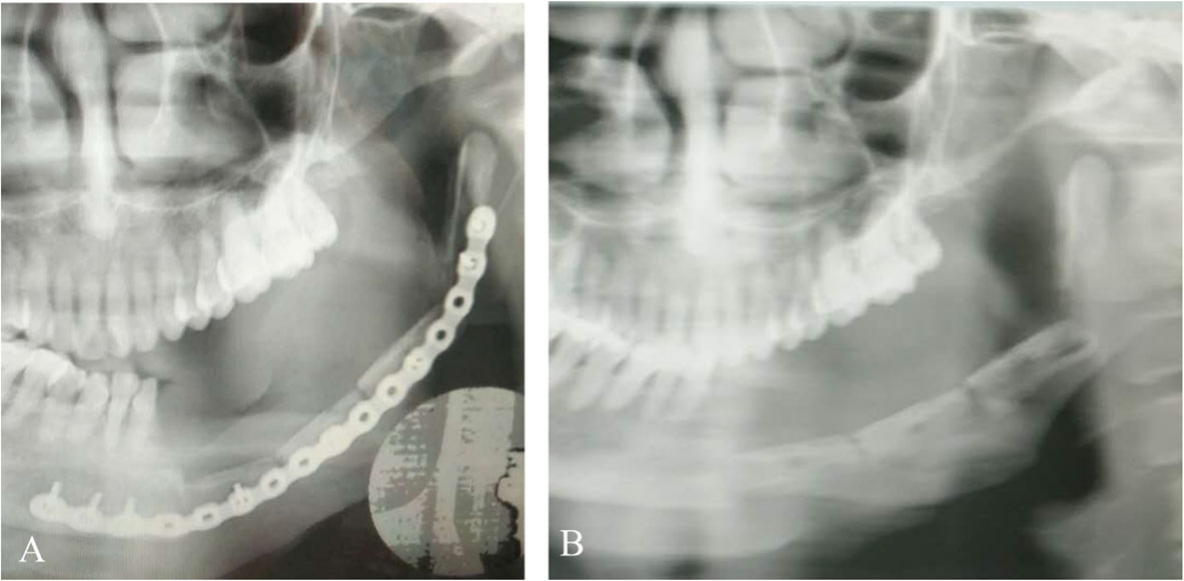
Panoramic radiograph showed that the non-vascularized fibular flaps survived after 12 months (**a**). The titanium plate was removed (**b**)

**Fig. 4:**
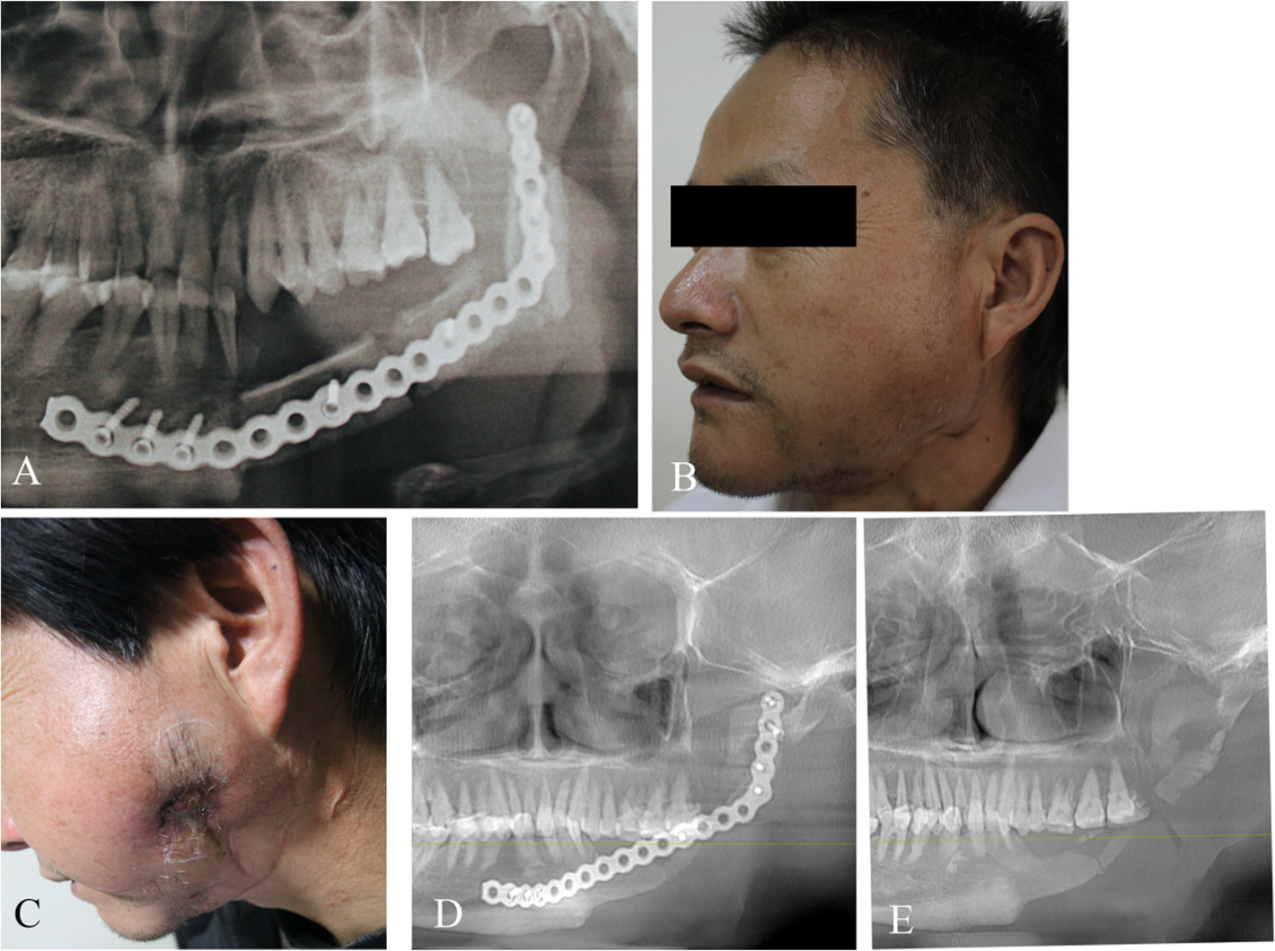
Panoramic radiograph revealed that the non-vascularized fibular flaps survived after 3 months (**a**). Postoperative view after 3 months (**b**). Long-term local chronic infection lasted for almost 4 years (**c**). Panoramic radiograph exhibited that the severe absorption occurred 6 years later (**e**). The titanium plate was removed (**e**)

In this study, 7 patients, including 5 males and 2 females with the age of 22–51 years (average, 33.7 years) developed postoperative vascular crisis, which was later confirmed as the mandibular ameloblastoma. In 3 patients, the vascularized fibular bone flaps healed favorably after surgical exploration, thanks to the early detection. Another 2 patients developed complete necrosis in the fibular bone flap; among them, one underwent surgical exploration, while the other one did not, and the fibular grafts were finally removed in the latter. The remaining two patients with non-vascular fibular bone flaps were followed up for 25 and 69 months, respectively. The titanium plate was removed in one of the patients 1 year later, while the other one suffered from postoperative local chronic infection for 2 years (Figs. 3 and 4). In this patient, the chronic infection was not controlled until the titanium plate was removed 6 years postoperatively due to severe fibular graft absorption (Fig. 4).

## Discussion

Costal bone and iliac bone may serve as the common donors of non-vascularized bone grafts for the reconstruction of mandibular defects when there is little or no soft tissue loss [2]. By contrast, the vascularized fibular flap is currently adopted for reconstructing large jaw defects, so as to restore the facial contour and chewing function of the patient, and this approach attains a high success rate, with the overall survival rate of up to 96–99% [12, 13]. Nonetheless, some cases still develop postoperative vascular crisis. Under such circumstances, early surgical exploration is of great significance to attain a successful operation. Notably, the delayed discovery or the presence of a more severe vascular crisis may lead to complete necrosis of the vascularized fibular flap. In this case, the transplanted fibular flap should be removed, and the mandibular defect should be treated by either immediate reconstruction with a vascularized bone flap, or bridging with a reconstruction plate prior to the subsequent mandibular defect reconstruction. However, this method is unfavorable to restore the facial contour and chewing function of patients. Besides, further reconstruction may not be feasible or desirable in some patients [12]. Consequently, it is a suitable temporary remedy to immediately change the vascularized fibular osteomyocutaneous flap with severe vascular crisis to the non-vascularized fibular graft.

In this study, two vascularized fibular flaps with severe vascular crisis were timely managed with non-vascularized fibular grafts, and they had survived at the 25 and 69 months of follow-ups. More importantly, the facial contours of these patients were maintained even though there was partial absorption. Typically, such success should be attributed to the early detection of postoperative vascular crisis, favorable preoperative physical condition, small range of soft tissue defect, and no history of radiotherapy in these patients. Coincidentally, Xiao et al. [14] had recently reported 10 cases with vascular crisis, and non-vascularized fibular bone grafts were applied in these patients to compensate for the failure of free vascularized iliac bone flaps. The above findings show that the non-vascularized fibular graft is promising to serve as a remediation technique after failure of vascularized fibular flap, which contributes to the successful restoration segmental mandibular defects and facial contours of patients. In fact, some studies report that bone defects are reconstructed by the non-vascularized iliac bone grafts, and most of these bone defects are derived from limb bones such as the poor healing of scaphoid fracture [15], ankle joint [16], discontinuous femoral neck fracture [17], and defect due to giant cell tumor in distal radius [18]. On the other hand, Jeong et al. [16] had successfully repaired one ankle joint with a 15-cm non-vascularized fibular flap. Moreover, Giordano et al. [19] also adopted a 21-cm non-vascular fibular flap to successfully repair the distal femoral condyle defect in an 86-year-old patient. All these successful cases mentioned above have indicated that it is feasible to reconstruct bone defects with the non-vascular fibula in clinical practice [20]. For instance, Lee et al. [21] and Wang et al. [22] had fixed the vascularized fibular flap to the mandibular base and placed the residual non-vascularized fibula onto the vascularized fibular flap to improve the height of the reconstructed mandible. These studies have partially suggested that, it is feasible to partially repair a mandibular defect with non-vascularized fibula, but it remains unclear about whether there is a significant difference between the planned non-vascularized fibula and the one changed based on a vascularized fibular flap due to severe vascular crisis. Nevertheless, in any case, the early detection of postoperative vascular crisis is the key to the success of this technique. Therefore, efforts should be made to guarantee a perforator flap in the vascularized fibular flap so as to facilitate postoperative observation. Among the 7 patients with vascular crisis in this study, two had no perforator flap (even during surgical exploration), but the fibular bone flap did not survive due to the lack of timely detection. However, in patients with L-shaped mandibular defects, the risk of venous crisis increases if the lower end of fibula is used to reconstruct the mandibular ramus where the perforator flap is located. Unfortunately, two patients in this study were encountered with venous crisis of the flap 2 days postoperatively, which was attributed to the above situation. Consequently, care should be taken to position the perforator flap and to avoid local compression in patients with L-shaped mandibular defects.

## Conclusions

It is a relatively suitable temporary remediation technique to immediately change a vascularized fibular osteomyocutaneous flap with postoperative severe vascular crisis to a non-vascular fibula graft, which may avoid complete necrosis and the subsequent serious adverse consequences.

## Data Availability

All data utilized in this study are not public but are available from the corresponding authors upon reasonable request.
